# Untargeted Metabolomics Analysis of Lactic Acid Bacteria Fermented *Acanthopanax senticosus* with Regard to Regulated Gut Microbiota in Mice

**DOI:** 10.3390/molecules29174074

**Published:** 2024-08-28

**Authors:** Yuanyuan Su, Xiang Fu, Pengwei Zhuang

**Affiliations:** 1 Chinese Materia Medica College, Tianjin University of Traditional Chinese Medicine, Tianjin 301617, China; 13018351625@163.com; 2 College of Agronomy and Agricultural Engineering, Liaocheng University, Liaocheng 252000, China; fuxiang2499570264@163.com

**Keywords:** lactic acid bacteria, fermentation, *Acanthopanax senticosus*, microbiota

## Abstract

Previous studies have shown that *Acanthopanax senticosus* (AS) has a beneficial preventive and therapeutic effect on colitis. The fermentation of lactic acid bacteria (LAB) can alter the efficacy of AS by modifying or producing new compounds with potential bioactive properties. However, the specific components and mechanisms that enhance the efficacy are still unclear. In the present experiment, untargeted metabolomics was used to analyze the changes in active components before and after LAB fermentation of AS. The aim was to explain the mechanism of AS fermentation in treating colitis using a colitis model in mice. The results indicated that the fermentation of LAB could enhance the levels of total flavonoids and total polyphenols in FAS. Additionally, the beneficial components such as Delphinidin chloride, Diosmetin, Psoralidin, and Catechol significantly increased (*p* < 0.05). The colitis treatment experiment demonstrated that fermented AS could alleviate symptoms and improve the morphology of colitis in mice by enhancing antioxidant enzymes like CAT, T-SOD, and T-AOC. It also regulated the composition and abundance of intestinal flora species, such as *Lactobacillus* and *Pseudogracilibacillus*. The effectiveness of fermented AS was significantly superior to that of unfermented AS (*p* < 0.05). In conclusion, this study contributes to the application of lactic acid bacteria in AS fermentation and reveals the mechanism of fermentation AS for colitis.

## 1. Introduction

The fermentation of herbal medicine with lactic acid bacteria (LAB) can enhance the content of active ingredients or generate new secondary metabolites [1]. Currently, the assessment of fermentation effects primarily relies on evaluating changes in beneficial components such as flavonoids, polyphenols, proteins, and others, as well as their biological activity [2]. After fermentation, the chemical composition of the fermentation product is complex, with low contents and a wide variety of components, making it challenging to identify all components using traditional detection methods.

The emergence of non-targeted metabolomics technology, which can detect the changes of compounds before and after fermentation, has the advantages of high-throughput technology, simplicity, high sensitivity, and high efficiency, making it an important tool to analyze the composition changes of fermentation compounds [3]. Du et al. analyzed the chemical composition changes after the solid-state fermentation of *Glycyrrhiza* stems and leaves using a widely targeted metabolomic analysis and found 461 differential metabolites, with 320 downregulated and 141 upregulated. The main types of differential metabolites were phenolic acids and flavonoids [4]. Li et al. used an untargeted metabolomics analysis to identify differential markers of two *Sorghum* varieties for baijiu fermentation, revealing 267 metabolites within both types of fermented *Sorghum*. Further analyses highlighted sphingolipids, 2,5-diketopiperazines, and methionine derivatives as critical markers for quality control [5]. Moreover, research indicated that fermented products have significant advantages in the treatment of gastrointestinal diseases [6,7].

Colitis is a non-specific inflammatory disease of the intestine that mainly affects the colonic tissue and submucosa [8]. Intestinal homeostasis disorder and oxidative stress are important triggers in colitis patients [9]. Previous studies have indicated the potential of LAB-fermented plant-based products in preventing or treating colitis by regulating intestinal flora and correcting metabolic disorders [10,11].

*Acanthopanax senticosus* (Rupr. & Maxim.) harms (AS) has a variety of bioactive components, such as phenolic acids, flavonoids, polysaccharides, saponins, and other phytochemicals [12]. Modern medical research has shown that AS possesses a wide range of biological activities, including antioxidant, anti-fatigue, anti-inflammatory, and immunomodulatory effects [13]. Gyoung et al. screened the anti-inflammatory compounds in AS fruit (*Ogaza*) extract through liquid chromatography-tandem mass spectrometry (LC-MS/MS). They identified 14 and 16 compounds in the negative and positive ion modes, respectively. For example, quercetin, hyperoside, acanthoside D, oleanolic acid, and scopoletin were identified as potential anti-inflammatory components [14]. Kim et al. demonstrated that the extrusion of AS leaves (ASLs) had protective effects on acute gastric mucosal lesions in rats by reducing inflammation and oxidative stress [15]. Ma et al. demonstrated that AS fermentation by *Lactobacillus rhamnosus* exhibited antioxidant activity and anti-inflammatory functions in crucian carp (*Carassius auratus*), leading to increased levels of CAT, GSH-PX, and SOD, as well as the upregulation of C3 and C4 concentrations [16]. Kim et al. found that the fermentation extract of the bark of AS exhibited anti-inflammatory activity in lipopolysaccharide-treated RAW 264.7 macrophage cells [17]. To date, there are no reports on whether the extracts of LAB-fermented AS could be more effective in relieving the symptoms of colitis.

In this context, the objective of this study was to investigate the influence of LAB-fermented AS on the active ingredient profile and therapeutic efficacy of colitis in mice. This experiment provides a comprehensive scientific foundation for the rational processing and effective utilization of fermented AS products.

## 2. Results

### 2.1. Changes in Active Ingredient Contents after Fermentation

**Figure 1:**
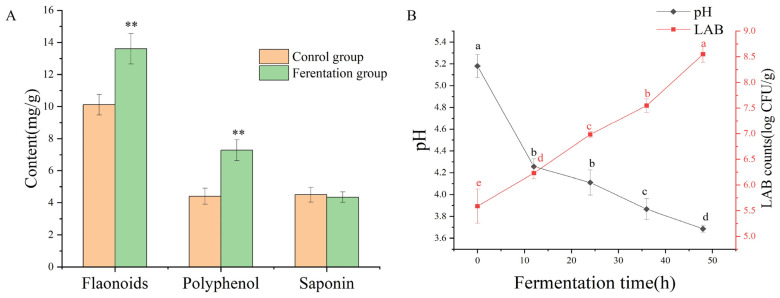
The contents change of active ingredient after fermentation (**A**); the change of pH value and viable cell counts during fermentation (**B**). ** represents a significant difference in plot (**A**) (*p* < 0.01). Different letters in different positions represent significant differences in plot (**B**) (*p* < 0.05).

By measuring the contents of polyphenols, flavonoids, and saponins after solid-state fermentation by LAB, it can be found that the contents of polyphenols and flavonoids increased significantly during the fermentation of AS (*p* < 0.05), while the contents of saponins decreased (*p* > 0.05), as shown in Figure 1A. The content of flavonoids was increased by 34.39% from 10.12 ± 0.64 mg of rutin equivalent (RE)/g dry weight (DW) of the sample to 13.60 ± 0.94 mg of RE/g DW. The content of polyphenols increased from 4.41 ± 0.50 mg of gallic acid equivalent (GAE)/g DW of the sample to 7.28 ± 065 mg of GAE/g DW, an increase of 65.08% (*p* < 0.01). The saponin content decreased from 4.50 ± 046 mg of ginsenosides equivalent (GE)/g DW to 4.35 ± 032 mg of GE/g DW, a decrease of 3.33% (*p* > 0.05).

### 2.2. Changes in Viable Cell Count and PH during the Fermentation Process

As shown in Figure 1B, the pH value rapidly decreased to 4.26 ± 0.07 within the first 10 h after fermentation. Subsequently, the rate of decrease slowed down, and the final pH value was 3.69 ± 0.03. The viable cell counts of LAB increased consistently throughout the fermentation period, with the LAB population ultimately increasing by 8.55 ± 0.16 log CFU/mL. As the number of LAB increased, the pH decreased. The trends in viable cell numbers were opposite to those of pH.

### 2.3. Untargeted Metabolomics Analysis

#### 2.3.1. Metabolic Profile Analysis

In positive ion mode, 1210 metabolites were identified, and 707 metabolites were identified in negative ion mode after solid-state fermentation of AS. The metabolites of positive and negative ion modes were combined and classified. The secondary classification of compounds according to the compound categories showed that the numbers of fatty acyls, prenol lipids, carboxylic acids and derivatives, organooxygen compounds, flavonoids, steroids and steroid derivatives, benzene and substituted derivatives, coumarins and derivatives, and phenols were 164, 158, 156, 123, 83, 62, 59, 36, and 34 types of compounds, respectively. The count plot of the compounds is shown in Figure S1.

#### 2.3.2. Metabolomics Multivariate Statistical Analysis

**Figure 2:**
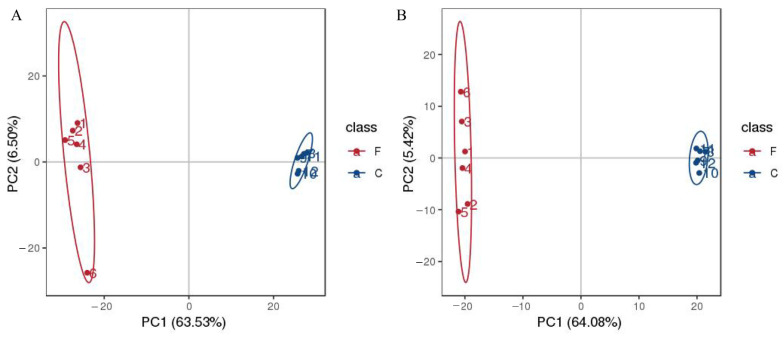
Principal component analysis (PCA) of the AS samples during the fermentation; in positive mode (**A**); in negative mode (**B**).

**Figure 3:**
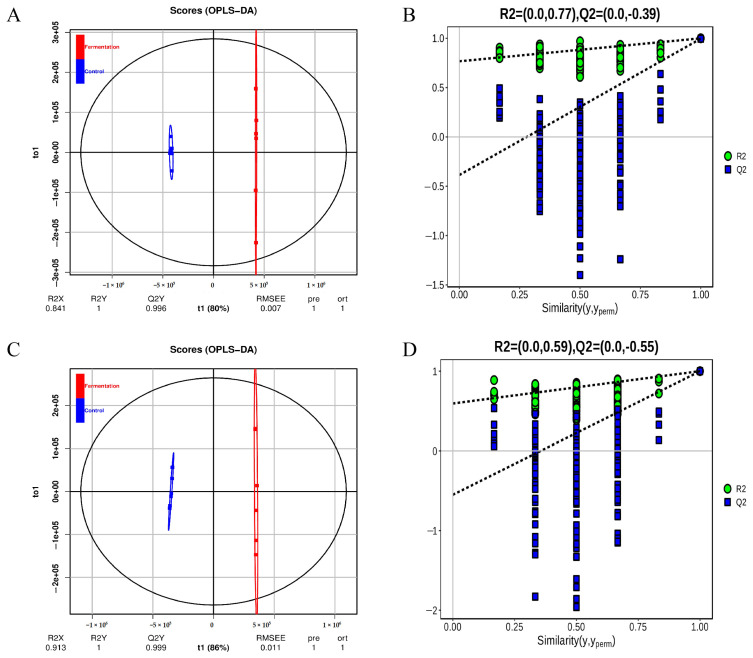
The diagram of orthogonal partial least-squares discriminant analysis (OPLS-DA) of metabolites (**A**), and permutation test plot (**B**) in positive ion modes; OPLS-DA diagram (**C**), permutation test plot (**D**) in negative ion modes.

As shown in Figure 2A, in positive mode, the PCA score plot showed 70.03% of the total variance (PC1, 63.53%; PC2, 6.50%). In negative mode (Figure 2B), the PCA score plot showed 69.5% of the total variance (PC1, 64.08%; PC2, 5.42%). The control group and fermentation group were clearly separated in each mode. The orthogonal partial least squares discriminant analysis (OPLS-DA) was used for the analysis of metabolites. The results show similar clustering patterns to the PCA. The OPLS-DA model evaluation parameters were R^2^ = (0.0, 0.77), Q^2^ = (0.0, −0.39) in positive mode, and R^2^ = (0.0, 0.59), Q^2^ = (0.0, −0.55) in negative mode (Figure 3A,C). Permutation testing showed that the model had good predictability and reproducibility without overfitting (Figure 3B,D).

#### 2.3.3. Differential Analysis of Key Metabolites during AS Fermentation

**Figure 4:**
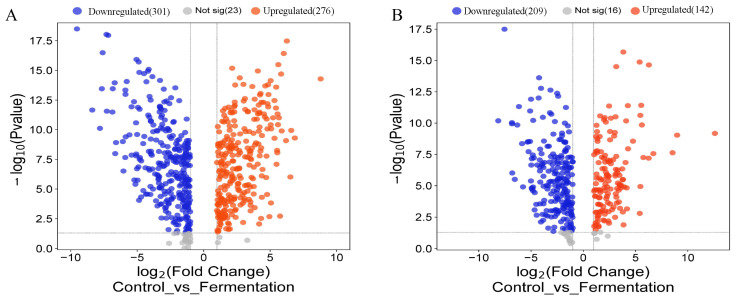
Differential metabolites during AS fermentation. Volcano plot of differential metabolites in pos mode (**A**) and in neg mode (**B**).

The screened differential metabolite signatures were visualized in the volcano plots during the fermentation process (Figure 4A,B). In positive mode, 600 compounds were identified as metabolites with significant differences (*p* < 0.05), 301 compounds were downregulated, and 276 compounds were upregulated. In negative mode, 367 compounds were identified as metabolites with significant differences (*p* < 0.05), 209 compounds were downregulated, and 142 compounds were upregulated (*p* < 0.05). For example, xanthurenic acid o-hexoside, 3-o-p-coumaroyl shikimic acid o-hexoside, hesperetin 5-o-glucoside, and xanthosine showed substantial downregulation, while 2,6-diaminooimelic acid, acetylcysteine, and phenylacetaldehyde exhibited remarkable upregulation.

#### 2.3.4. Metabolic Pathway Analysis

**Figure 5:**
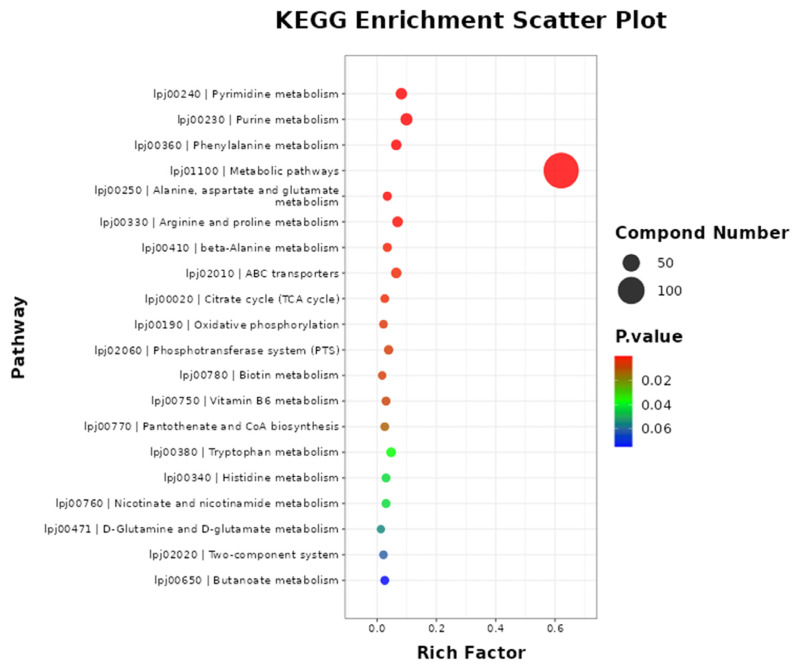
The metabolic pathway enrichment of differential metabolites. Note: the metabolites pathway of differential metabolites was enriched using MBROLE 2.0: (http://csbg.cnb.csic.es/mbrole2/index.php) (accessed on 1 March 2024). The top 20 principal pathways were chosen for study with a significance level of *p* < 0.05.

As shown in Figure 5, metabolic pathways were the most enriched pathways, which included 144 differential metabolites (*p* < 0.001). The metabolic pathways of purine metabolism, pyrimidine metabolism, arginine and proline metabolism, phenylalanine metabolism, ABC transporters, and tryptophan metabolism included 23, 19, 16, 15, 15, and 11 metabolites, respectively.

### 2.4. Effect of FAS Extracts on DSS-Induced Colitis in Mice

**Figure 6:**
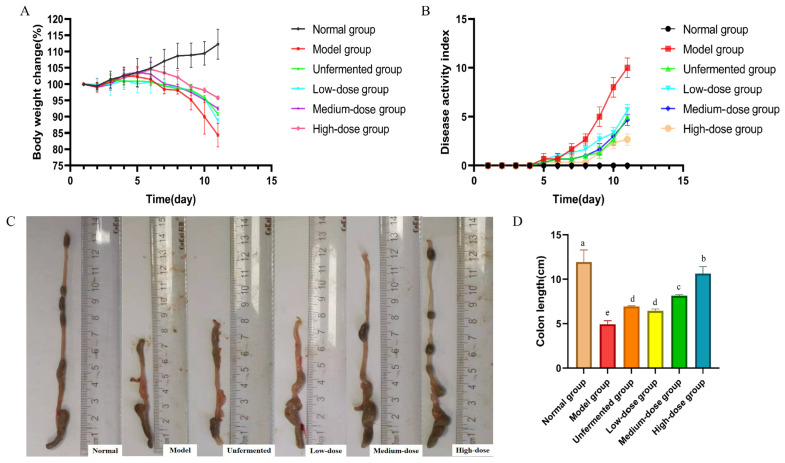
Effects of FAS on colitis in mice. Body weight change. (**A**) DAI score during experimental colitis. (**B**) Images of the colon length. (**C**) Colonic length in mice. (**D**) Data are presented as mean ± SD, n = 6. Different letters represent significant difference on the different column (*p* < 0.05).

As shown in Figure 6A, compared with the model group, the FAS treatment groups were able to alleviate weight loss in mice with a dose-dependent effect. The body weight loss of mice in the medium- and high-dose treatment groups of FAS was lower compared to the unfermented group. In all treatment groups, the disease activity index (DAI) scores decreased compared to the model group, with the highest reduction observed in the high-dose group of FAS (Figure 6B). As depicted in Figure 6C,D, the length of the colon was significantly longer in the FAS medium- and high-dose groups than in the model group (*p* < 0.001).

### 2.5. Histological Effects of FAS on Colitis

**Figure 7:**
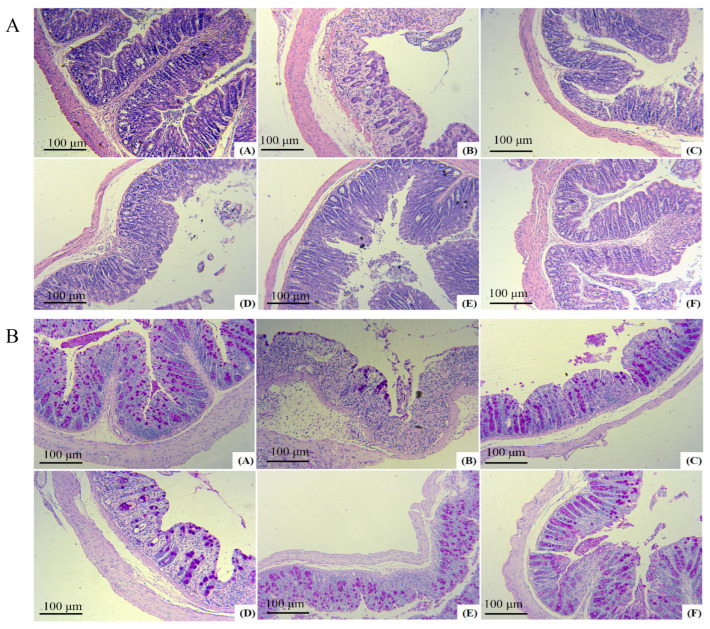
Protective effects of FAS on the colon. H&E staining images of each **group A**. PAS staining images of each **group B**. Note: (**A**) represents the normal group, (**B**) represents the model group, (**C**) represents the unfermented group, (**D**) represents the low-dose group, (**E**) represents the medium-dose group, (**F**) represents the high-dose group.

As shown in Figure 7A,B, it could be seen that the colonic mucosal tissues of mice in the model group were severely damaged, with the absence of glands, crypts, and cup cells, as well as a large number of inflammatory factors overflowing and a more severe cellular infiltration phenomenon. After different doses of FAS intervention, the damage in the colons of mice was reduced to varying degrees. The low-dose group showed less improvement in colitis than the model group, with only a small number of cupped cells present, and a low number of glands. The medium-dose group exhibited a more noticeable improvement in the colonic mucosa. Some cupped cells were arranged orderly, the number of glands was angular but unevenly distributed, and the overall structural damage was mild. However, the inflammatory cell infiltration was still more apparent. Colonic symptoms in the high-dose group were mild, mainly manifested by a more intact mucosal structure, partial detachment of mucosal epithelial cells, a slight infiltration of inflammatory cells around the glands, and a slight reduction in cupped cells.

### 2.6. Biochemical Analysis of the Serum

**Figure 8:**
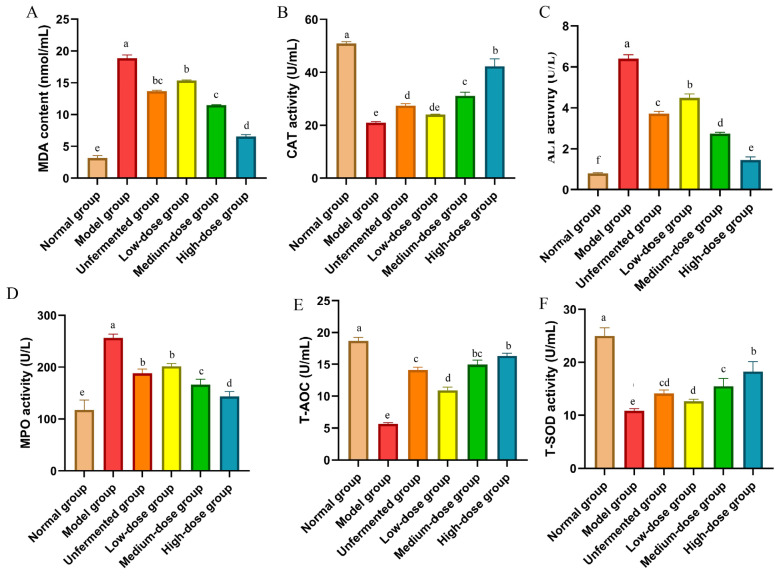
Effects of FAS on expressing antioxidant parameters in the colon in DSS-induced colitis mice. (**A**) MDA. (**B**) CAT. (**C**) ALT. (**D**) MPO. (**E**) T-AOC. (**F**) T-SOD. Data are presented as mean ± SD, n = 6. Different letters represent significant difference on the different column (*p* < 0.05).

The levels of Alanine aminotransferase (ALT), catalase (CAT), malondialdehyde (MDA), myeloperoxidase (MPO), total antioxidant capacity (T-AOC), and total superoxide dismutase (T-SOD) were determined using a spectrophotometer following the experimental instructions provided by Nanjing Jiancheng Biological Engineering Research Institute Co., Ltd., Nanjing, China. The significant increase in MDA, ALT, and MPO contents in the serum of mice in the model group compared to that of the normal group (*p* < 0.05) was observed. Following treatment with AS and FAS, the MDA, ALT, and MPO contents significantly decreased in all treatment groups, with the most notable reduction seen in the high-dose group (*p* < 0.05). The CAT, T-AOC, and T-SOD activities were significantly reduced in the model group, while treatment with AS and FAS increased CAT, T-AOC, and T-SOD activities in all treatment groups. The most significant effect was observed in the high-dose group (*p* < 0.05). The effects of the medium- and high-dose groups were more efficient than those of the unfermented group (*p* < 0.05) (Figure 8).

### 2.7. Influence of FAS on Bacterial Diversity

**Figure 9:**
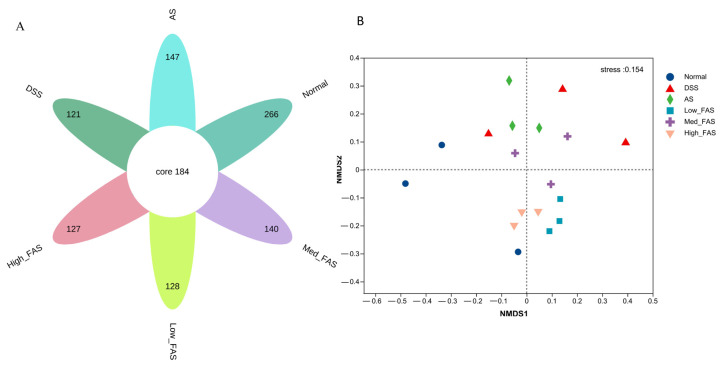
The effects of FAS on the diversity of gut microbiota of DSS-induced colitis in mice. (**A**) Flower plot showing OTUs that differed in each group; (**B**) NMDS score plot showing the overall structure of gut microbiota.

A flower plot was created using the results of operational taxonomic units (OTUs) to compare the similarity of OTUs at different taxonomic levels. Figure 9A shows that 184 OTUs were shared by all groups. Additionally, 266, 121, 147, 128, 140, and 127 OTUs were uniquely present in the normal group, model group, unfermented group, low-dose group, medium-dose group, and high-dose group, respectively. The normal group had the highest number of OTUs, and the model group had the lowest number of OTUs.

As shown in Table 1, the Chao index, Ace index, and Shannon index in the model group were significantly lower than in the normal group (*p* < 0.05). The treatment groups increased the values of the Chao index, Ace index, and Shannon index. Additionally, the Chao index in the model group, and the Ace index and Shannon index in the medium-dose group were significantly higher than those in the model group (*p* < 0.05). The Simpson index in the model group was higher than in the other groups, but the difference was not significant (*p* > 0.05).

The NMDS analysis revealed a significant alteration in the structure of the gut microbiota in the model group. Following treatment with FAS and AS, a notable shift towards the normal group in the gut microbiota was observed (Figure 9B), with the high-dose group showing closer proximity to the normal group.

**Figure 10:**
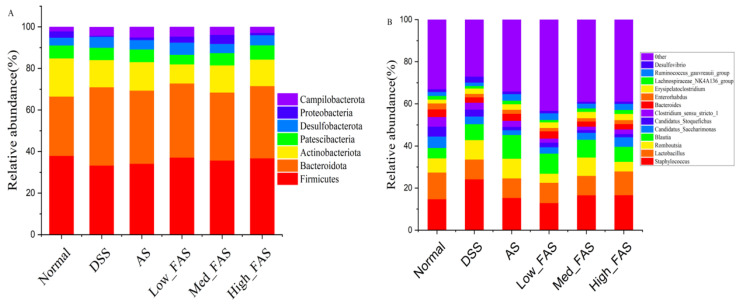
FAS altered gut microbiota in mice with DSS-induced colitis. Gut microbiota abundance at the phylum level (**A**). Gut microbiota abundance at the genus level (**B**).

At the phylum level (Figure 10A), the composition of the phylum in the intestine was dominated by *Firmicutes*, *Bacteroidota*, *Actinobacteriota*, and *Patescibacteria*, which accounted for approximately 90% of the total abundance of the normal, DSS, AS, low_FAS, med_FAS, and high_FAS groups. The structure of the flora was similar among the groups, but the proportion of the composition varied greatly. Different doses of FAS had varying effects on the intestinal flora. The relative abundance of *Bacteroidota* in the DSS group increased compared to the normal group. Simultaneously, FAS treatment suppressed the trend of increasing *Bacteroidota* relative abundance and increased the relative abundance of *Firmicutes* in the intestine. At the genus level (Figure 10B), *Staphylococcus* and *Lactobacillus* were the two most abundant genera. The relative abundance of *Staphylococcus* was increased in the DSS group compared to the normal group, while the relative abundance of *Lactobacillus* decreased in the DSS group compared to the normal group. The AS and FAS altered the changes to some extent.

### 2.8. The Influence of FAS on the Gut Microbiome

**Figure 11:**
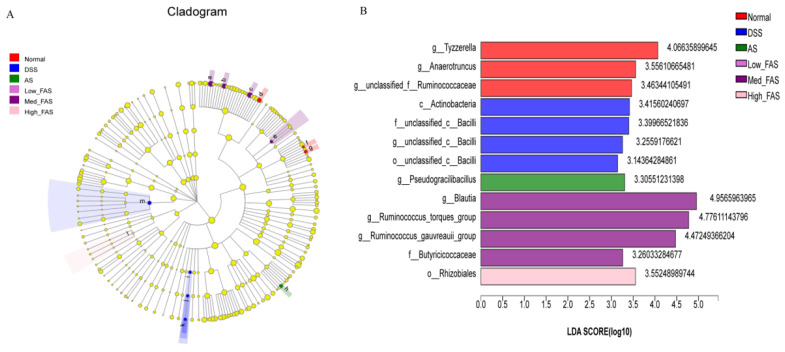
Effects of FAS on the gut microbiota diversity. LEfSe analysis (**A**). LDA scores of the differentially abundant taxa (**B**). Note: a: g_Blautia; b: g_Ruminococcus_gauvreaui group; c: g_Ruminococcus_torques_group; d: g_Tyzzerella; e:f_Butyricicoccaceae; f: g_Anaerotruncus; g: g_unclassified_f_Ruminococcaceae; h: g_Pseudogracilibacillus; i: o_unclassified_c_Bacilli; j: f_unclassified_c_Bacilli; k: g_unclassified_c_ Bailli; l: o_Rhizobiales; m: c_Actinobacteria.

The experimental results were also validated using a linear discriminant analysis (LDA), which showed that in Figure 11B, more significant LDA scores represented a greater effect of species abundance on the differential effect (*p* < 0.05). In the DSS group, *Bacilli* and *Actinobacteria* were identified as the dominant intestinal flora. *Anaerotruncus*, *Tyzzerella*, and *Ruminococcaceae* were the key phylotypes of the gut microbiota in the normal group. *Pseudogracilibacillus* was the dominant species in the AS group. *Pseudogracilibacillus*, *Blautia*, *Ruminococcus_torques_group*, *uminococcus_gauvreauii_group*, *Butyricoccaceae*, and *Rhizobiales* were the dominant species in different concentration groups of FAS, respectively.

## 3. Discussion

Fermentation is one of the most widely used procedures, and it is constantly evolving as a technology for improving the nutritional value of plant-based food [18]. Much scientific evidence has demonstrated the high nutritional value and health benefits of fermented foods compared to the unfermented matrix. For example, they can increase immune defenses, have anti-inflammatory and antioxidant properties, and alleviate symptoms in colitis [19]. Present experiments have shown that components such as flavonoids and polyphenols are significantly increased when lactic acid bacteria ferment AS. This result is consistent with most of the literature [20,21]. The increase in polyphenols and flavonoids may be due to the conversion of bound polyphenols and flavonoids into free forms by lactic acid bacteria fermentation [22]. The decrease in saponin content may be attributed to some saponins being hydrolyzed [23].

Non-targeted metabolomics was used to investigate how LAB fermentation affects small molecule metabolites in AS. The results indicate that LAB fermentation substantially influences the components of AS, with 418 components increasing and 510 components decreasing. Among the differential metabolites, multiple compounds that benefit health and intestinal care were found. In particular, flavonoids with polyphenols increased the most. Non-targeted metabolomics studies showed that Delphinidin chloride increased by 5.41-fold, Diosmetin by 4.48-fold, Psoralidin by 4.37-fold, and Catechol by 48.78-fold, etc., and significantly increased in content compared to pre-fermentation. Catechins are natural polyphenols with antioxidant properties and have a variety of pharmacological effects, including anti-inflammatory, antioxidant, and antiviral effects [24]. Delphinidin chloride, a flavonoid compound, can modulate the JAK/STAT3 and MAPK signaling pathways and induce apoptosis in tumor cells [25].

Non-targeted metabolomics studies have shown that many anti-inflammatory and antimicrobial compounds undergo a significant increase after the fermentation of AS. For instance, Loxoprofen has increased by 14.28-fold after fermentation and is commonly used as an anti-inflammatory drug [26]. Nitrofurantoin content increased from 0.0305 before fermentation to 1.4692 after fermentation, which is an antibiotic compound [27]. Furthermore, 3-Phenyllactic acid showed a significant increase after fermentation (87-fold), making it an effective bacteriostatic drug [28]. Similar compounds that significantly increase after fermentation include 2,5-Dihydroxybenzaldehyde, DL-4-Hydroxyphenyllactic acid, Bialaphos, and Sorbic acid.

Additionally, in the untargeted metabolomics assay, we also observed a notable increase in compounds such as Cordycepin, Monotropein, Orientin, Engeletin, and Vitamin C. All these compounds are biologically active substances that benefit the health of the organism. Studies have shown that cordycepin alleviates inflammatory responses and oxidative stress in a variety of cells and regulates mitochondrial function [29]. Monotropein exerts its anti-tumor effects primarily by inhibiting Bcl-2 and increasing Bax, inducing G1–S cycle arrest in colorectal cancer. It might play an anti-cancer role through the Akt signaling pathway [30]. Orientin is a class of flavonoid carbon glycosides with pharmacological effects such as cardiovascular protection, anti-aging, anti-tumor, anti-inflammatory, and blood sugar regulation [31]. Therefore, the fermentation treatment can increase the content of numerous beneficial components in AS, which is advantageous for treating colitis in mice.

In this experiment, DSS-induced colitis model mice were used to evaluate the anti-inflammatory effect of FAS. Weight loss, loose stools, bleeding, and diarrhea were all observed in mice in the DSS-induced colitis model group, indicating that the mouse model of colitis has been successfully established. The AS and FAS treatments significantly ameliorated DSS-induced weight loss, diarrhea, and shortened colon in mice, indicating that AS and FAS have a therapeutic effect on colitis in mice. Furthermore, the treatment effect in the FAS medium-dose and FAS high-dose groups was better than that of the AS group.

Oxidative stress has been attributed as a pathogenic factor of colitis [32]. Antioxidant enzymes such as SOD, CAT, and AOC are severely decreased, while oxidative stress metabolites such as MDA and MPO show a significant rise. These changes have been linked to the onset and manifestations of colitis [33]. Many pieces of evidence indicate that relieving oxidative stress is useful in the treatment of UC [34]. In the present study, FAS significantly increased the levels of CAT, T-AOC, and T-SOD, and inhibited the levels of MDA, MPO, and ALT in DSS-induced colitis mice. Studies have shown that AS is effective in treating colitis because it is rich in flavonoids and polyphenols [35]. Flavonoids and polyphenols have good antioxidant activity, can relieve the symptoms of colitis, and can regulate the balance of intestinal flora [36,37]. In the present study, FAS had a higher content of flavonoids and polyphenols, which was more beneficial for the treatment of colitis.

The application of high-throughput and metagenetic sequencing technologies has led to the possibility of identifying and analyzing intestinal flora [38]. Many studies have shown that intestinal flora plays a role in the metabolism of nutrients, intestinal mucosal immunity, endocrine functions, and more [39]. The homeostasis of intestinal flora is crucial for the body’s health, garnering increasing attention from researchers and becoming a target for intervention in diseases [40]. Research on intestinal flora disorders in the pathogenesis and development of colitis is expanding. Data also indicate that several medicines, such as palmatine [41] and Valeriana jatamansi [42], can alleviate colitis by regulating intestinal flora.

Our study is consistent with the results of many studies. The balance of intestinal flora in colitis mice was obviously disturbed, and treating mice with FAS restored flora balance, which increased the number of OTUs and the degree of alpha diversity. However, the relative abundance of harmful bacteria (e.g., *Escherichia*, *Erysipelotrichaceae*, *Staphylococcus*, etc.) was decreased, and the relative abundance of beneficial bacteria (e.g., *Lactobacillus*, *Parabacteroides*, *Lachnospiraceae*, etc.) was increased. *Lachnospiraceae* functions as a probiotic by hydrolyzing starch and other sugars to produce butyric acid and other short-chain fatty acids [29]. Its abundance increased in the FAS group. The probiotic *Lactobacillus* also increased in the FAS-treated group. *Erysipelotrichaceae* is a harmful bacterium capable of causing arthritis in infected animals and symptoms of dengue [30]. Compared with the DSS group, FAS treatment reduced the abundance of *Erysipelotrichaceae*. Therefore, FAS has the potential to regulate the balance of the intestinal microbiota to alleviate the symptoms of colitis.

## 4. Materials and Methods

### 4.1. Animal

Sixty SPF male ICR rats, 5 weeks old and weighing 30 ± 2 g, were purchased from Jinan Pengyue Laboratory Animal Breeding Co., Ltd. (Jinan, China).

All animal experiments were conducted in accordance with the guidelines of the Animal Center of China. The ethics committee approved the animal experiment by the Institutional Animal Care and Use Committee of Liaocheng University (No: 2023022732).

### 4.2. LAB Strain and Growth Conditions

LAB were obtained from laboratory preservation strains, which were identified as *lactobacillus pentosus*. LAB were inoculated in De Man, Rogosa, and Sharpe (MRS) medium and incubated at 37 °C for 24 h. The concentration of bacteria was determined using a spectrophotometer at 600 nm.

### 4.3. Cellulase Hydrolysis of AS before Fermentation

A sterile conical flask was used to perform the hydrolysis of AS. A mass of 50 g of dried AS powder was accurately weighed and transferred to the conical flask. Citric acid buffers (0.01 M, pH 5.0) were used to regulate the pH and water content. The pH value was adjusted to 5.0, and the water content was regulated to 75%. Cellulase (10,000 units) was added to the AS powder for enzymatic digestion. Subsequently, the conical flask was placed in a water bath at 50 °C for 12 h, followed by sterilization for 20 min at 121 °C to inactivate enzymes and sterilize the solution.

### 4.4. LAB Fermentation of AS and Extracts Preparation

The mixing ratio of substrates was chosen based on the preliminary experiments. The sterilized samples were supplemented with 5% sucrose and inoculated with LAB at 1 × 10^7^ CFU/g, before being hermetically sealed. Subsequently, the mixture was placed in an incubator at 37 °C for 48 h. Throughout the fermentation process, samples were collected at various time points (0, 12, 24, 36, and 48 h) for further analysis. All conditions were the same in the control group, except for the absence of LAB inoculation. All the samples were tested in triplicate to ensure reproducibility. The extraction of unfermented AS and FAS samples was conducted using an ultrasonic-assisted extraction method. The extraction conditions were as follows: 60% ethanol as the extraction solvent, ultrasonic time of 40 min, ultrasonic power of 600 W, extraction temperature of 60 °C, and a material–liquid ratio of 1:30 (g: mL). After extraction, the extract was cooled, filtered, and centrifuged at 10,000 rpm for 10 min. The supernatant was collected, concentrated, and then freeze-dried for sequencing animal assays.

### 4.5. Measurement of the Active Ingredients

To compare the changes in the content of the active components of AS before and after fermentation, the phenol, the flavonoid, and saponin contents were determined. Phenol content was evaluated using the colorimetric Folin–Ciocalteu method, with Gallic acid as the standard. The flavonoid content was determined by spectrophotometry using the NaNO_2_-Al(NO_3_)_3_-NaOH system, with rutin as the reference. Saponin content was determined using the vanillin-sulfuric acid chromogenic method, with ginsenosides as the standard. Detailed measurement methods are described by Liu et al. [43].

### 4.6. Determination of LAB Viable Cell Counts and pH

Viable cell counts of LAB were determined using a standard plate counting method. A FE28 standard digital pH meter was utilized to measure the pH of the samples at various stages of the fermentation process [44].

### 4.7. Analysis of the Metabolite Profiles by Untargeted Metabolomics

The metabolite profiles of AS before and after fermentation were analyzed using untargeted metabonomic technology, and the differential metabolites were determined through multivariate statistical analysis methods. The extract was dissolved in an 80% methanol solution in water (*v*/*v*), then centrifuged at 15,000× *g* for 20 min at 4 °C. The supernatant was collected for subsequent experiments. UHPLC-MS/MS assays and services were conducted and supported by Wekemo Tech Group Co., Ltd. in Shenzhen, China. The procedures and steps are provided in the Supplementary Materials.

### 4.8. Animal Experiment of DSS-Induced Colitis in Mice

The mice were randomly divided into 6 groups (n = 6): a blank control group, a DSS model group, an AS group (200 mg/mL), a DSS + low FAS group (100 mg/mL), a DSS+ middle FAS group (200 mg/mL), and a DSS+ high FAS group (400 mg/mL), with 10 animals in each group. Each group was administered a dose of 10 mg/kg body weight via gavage for 11 d. The normal and DSS groups received saline via gavage; the AS group received an unfermented AS extract solution; and the FAS low, middle, and high groups received different concentrations of FAS extracts. Throughout the experiment, all groups were provided with purified drinking water from day 1 to day 3. From day 4 to day 10, the mice in the blank group continued to drink plain water, while the remaining groups were given a 3% DSS aqueous solution.

### 4.9. Assessment of Daily Disease Activity and Sample Collection

During the test period, the weight of the mice was recorded, and stool properties were observed. Fecal occult blood was detected using a hydrogen peroxide reagent. The Disease Activity Index (DAI) scores were calculated [45]. The scoring criteria are presented in Table S1. On day 11, the mice were euthanized. Their colons were promptly dissected and laid flat on a dissecting board to measure their length. Rectal fecal samples were collected in sterile E.P. tubes and frozen at −80 °C. Subsequently, blood samples were collected in the E.P. tubes and centrifuged at 5000 g for 5 min at 4 °C. The serum was then transferred to a new E.P. tube and stored at −80 °C.

### 4.10. Histological Changes of Colon Tissue

Colon specimens were fixed using 4% (*w*/*v*) paraformaldehyde for 24 h. Subsequently, the samples were paraffin-embedded, and 4 µm sections were sliced and stained with hematoxylin and eosin (HE) as well as periodic acid-Schiff stain (PAS), which was performed by Wuhan Servicebio Technology Co., Ltd. (Wuhan, China). The histopathological changes were examined under a light microscope.

### 4.11. Biochemical Analysis of Serum

The blood was collected from the eyes of mice and centrifuged at 5000 rpm for 5 min at 4 °C to obtain the serum. The levels of MDA, MPO, T-SOD, ALT, and T-AOC were measured in the serum using the spectrophotometric method with commercial kits from Nanjing Jiancheng Bioengineering Institute. The test was performed according to the instructions in the kit package insert. The OD value was determined by using a spectrophotometer (UV-8000, Yuanxin, Shanghai, China).

### 4.12. Gut Microbiota Analysis

The fecal samples were sent to Majorbio Bio-Pharm Technology Co., Ltd. (Shanghai, China) for 16S rRNA gene sequencing. Initially, DNA was extracted from the fecal samples using a DNA kit. The V3–V4 region of the bacterial 16S rRNA gene was amplified with primer pairs 338F (5′-ACTCCTACGGGAGGC AGCAG-3′) and 806R (5′-GGACTACHVGGGTWTCTAAT-3′). Sequencing was carried out using an Illumina MiSeq PE300 platform (Illumina, San Diego, CA, USA) in Majorbio Bio-Pharm Technology Co., Ltd. (Shanghai, China). The sequencing data processing and bioinformatics analysis were performed on the Majorbio, Cloud Platform, a free online platform (https://www.majorbio.com) (accessed on 1 March 2024).

### 4.13. Statistical Analysis

The statistical analysis was performed using GraphPad Prism 8.0 software. The data were expressed as mean ± standard deviation, and comparisons between groups were conducted using one-way ANOVA and LSD tests.

## 5. Conclusions

This study compared the composition changes of compounds before and after fermentation using untargeted metabolomics. It also contrasted the effects of fermented and unfermented compounds on a colitis mouse model. The experimental results indicate a significant increase in the content of beneficial flavonoids and saponins after fermentation. In a colitis mouse model, the treatment effect of fermented AS is superior, with studies demonstrating an increase in antioxidant capacity and a balance in the intestinal flora, suggesting its potential therapeutic mechanism, consistent with the pharmacological effects of flavonoids and polyphenols. The findings of this study also provide new insights for using LAB in fermenting medicinal plants. The next step involves investigating the mechanisms of change and the clinical effects of specific compounds that undergo significant changes during fermentation.

## Figures and Tables

**Table 1:** Alpha delivery analysis of the gut microbiota.

Groups	Chao Index	Ace Index	Shannon Index	Simpson Index
Normal group	374.612 ± 20.452	377.728 ± 18.905	3.784 ± 0.252	0.156 ± 0.010
Model group	270.764 ± 27.847 *	264.545 ± 29.295 *	2.607 ± 0.183 *	0.217 ± 0.067
Unfermented group	324.990 ± 27.930 #	279.244 ± 4.641	2.968 ± 0.143	0.183 ± 0.012
Low-dose group	317.256 ± 49.226	289.361 ± 20.983	2.916 ± 0.165	0.164 ± 0.023
Medium-dose group	284.126 ± 13.553	320.810 ± 37.523 #	3.243 ± 0.311 #	0.160 ± 0.026
High-dose group	297.684 ± 10.418	305.646 ± 35.677	2.901 ± 0.348	0.165 ± 0.035

* *p* < 0.05, model group compared to the normal group. # *p* < 0.05, the treatment groups compared to the model group.

## Data Availability

Data will be made available on request.

## Supplementary Materials

The following supporting information can be downloaded at: https://www.mdpi.com/article/10.3390/molecules29174074/s1. Figure S1: The count plot of the compounds; Table S1: The score of DAI; Section S3: UHPLC-MS/MS analysis. The accession number(s) of the 16S rRNA sequence can be found at: https://www.ncbi.nlm.nih.gov/sra/PRJNA1148376 (accessed on 15 August 2024).
