# Supplementary material for: Untargeted Metabolomics Analysis of Lactic Acid Bacteria Fermented Acanthopanax senticosus with Regard to Regulated Gut Microbiota in Mice
Source: Molecules. 2024 Aug 28;29(17):4074. doi: 10.3390/molecules29174074 (PMC11396594; doi:10.3390/molecules29174074)
Supplement: Supplementary file 1 [file molecules-29-04074-s001.zip › molecules-3147637-supplementary.pdf]

Untargeted metabolomics analysis of lactic acid bacteria fermented *Acanthopanax senticosus with regard to regulated gut microbiota* in mice

S1. The score of DAI

Table S1 The score of DAI

Score	Weight loss（%）	Stool consistency	Blood stool
0	0	Normal	Normal
1	1~5	Soft but still formed	
2	5~10	Very soft	Occult bleeding
3	10~15	Sticky	
4	15~	Diarrhea	Dominant bleeding

S2. The count plot of the compounds

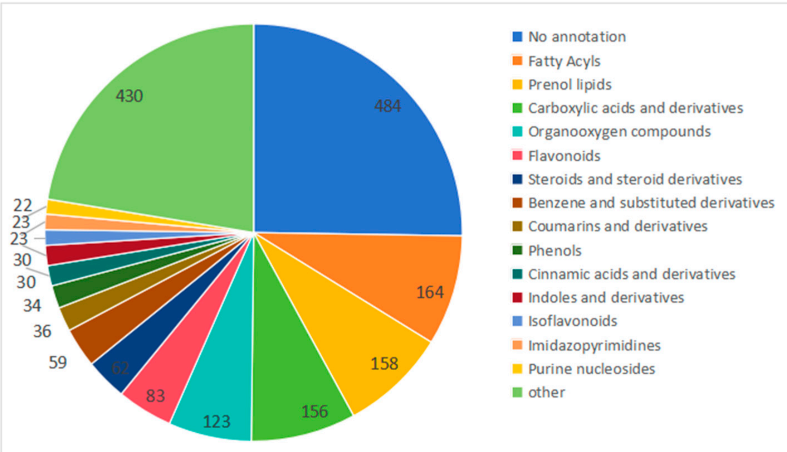

Figure S1 The count plot of the compounds

S3. *UHPLC-MS/MS Analysis*

S3.1 UHPLC-MS/MS Analysis

UHPLC-MS/MS analyses were performed using a Vanquish UHPLC system (ThermoFisher, Germany) coupled with an Orbitrap Q Exactive TMHF mass spectrometer or Orbitrap Q Exactive TMHF-X mass spectrometer (Thermo Fisher, Germany). Samples were injected onto a HypersilGoldcolumn (100×2.1mm,1.9μm) using a 12-min linear gradient at a flow rate of 0.2mL/min. The eluents for the positive and negative polarity modes were eluent A (0.1% FA in Water) and eluent B (Methanol). The solvent gradient was set as follows: 2% B, 1.5 min; 2-85% B, 3 min; 85-100% B, 10 min; 100-2% B, 10.1 min; 2% B, 12 min. The Q Exactive

TM HF mass spectrometer was operated in positive/negative polarity mode with a spray voltage of 3.5 kV, capillary temperature of 320°C, sheath gas flow rate of 35 psi and aux gas flow rate of 10 L/min, S-lens RF level of 60, and aux gas heater temperature of 350°C.

3.2 Data processing and metabolite identification

The raw data files generated by UHPLC-MS/MS were processed using the Compound Discoverer 3.3 (CD3.3, ThermoFisher) to perform peak alignment, peak picking, and quantitation for each metabolite. The main parameters were set as follows: the peak area was corrected with the first QC; actual mass tolerance, 5 ppm; signal intensity tolerance, 30%; and minimum intensity, et al. After that, peak intensities were normalized to the total spectral intensity. The normalized data were used to predict the molecular formula based on additive ions, molecular ion peaks, and fragment ions. Then, peaks were matched with the mzCloud (https://www.mzcloud.org/)，mzVaultand MassListdatabase to obtain the accurate qualitative and relative quantitative results. Statistical analyses were performed using the statistical software R (R version R-3.4.3), Python (Python 2.7.6 version), and CentOS (CentOS release 6.6) , and when data were not normally distributed, they were standardized according to the formula: sample raw quantitation value / (The sum of sample metabolite quantitation value / The sum of QC1 sample metabolite quantitation value) to obtain relative peak areas. Compounds whose CVs of relative peak areas in QC samples were greater than 30% were removed, and finally the metabolites' identification and relative quantification results were obtained.
